# The long-term prognostic value of serum 25(OH)D, albumin, and LL-37 levels in acute respiratory diseases among older adults

**DOI:** 10.1186/s12877-022-02836-8

**Published:** 2022-02-21

**Authors:** Matti Aronen, Laura Viikari, Henriikka Langen, Ia Kohonen, Maarit Wuorela, Tytti Vuorinen, Maria Söderlund-Venermo, Matti Viitanen, Carlos Arturo Camargo, Tero Vahlberg, Tuomas Jartti

**Affiliations:** 1 Department of Geriatrics, Turku University Hospital, Turku, Finland; 2 Uikunkuja 7, N28100 Pori, Finland; 3 Turku City Hospital, Turku, Finland; 4 Department of Radiology, Turku University Hospital, Turku, Finland; 5 Department of Medical Microbiology, Turku University Hospital and Institute of Biomedicine, University of Turku, Turku, Finland; 6 Department of Virology, University of Helsinki, Helsinki, Finland; 7 Massachusetts General Hospital, Harvard Medical School, Boston, USA; 8 Department of Biostatistics, University of Turku and Turku University Hospital, Turku, Finland; 9 PEDEGO Research Unit, Medical Research Center, University of Oulu, Oulu, Finland; 10 Department of Pediatrics and Adolescent Medicine, Oulu University Hospital, Oulu, Finland; 11 Department of Pediatrics and Adolescent Medicine, University of Turku and Turku University Hospital, Turku, Finland

**Keywords:** Older adults, Etiology, Albumin, LL-37, 25(OH)D, Respiratory virus

## Abstract

**Background:**

Older adults are more susceptible to respiratory tract infection than healthy working age adults. The increased susceptibility of older adults is thought to be interlinked with vitamin D status, nourishment, and immunological state in general. Data are scarce whether these parameters could serve as prognostic markers.

**Aim:**

To study whether serum 25(OH)D, albumin, and LL-37 level could give prognostic value of long-term survival in the older adults with multimorbidity and acute respiratory infection.

**Methods:**

Consecutive episodes of hospital care of patients 65 years and older with respiratory symptoms were prospectively studied as a cohort. Standard clinical questionnaire was filled by the study physician. Laboratory markers included serum levels of 25(OH)D, albumin and LL-37, C-reactive protein (CRP), white blood cell count (WBC) and polymerase chain reaction diagnostics for 14 respiratory viruses. Pneumonia was confirmed by chest radiographs. Respiratory illness severity, death at ward, length of hospital stays, and 5-year survival were used as outcomes.

**Results:**

In total, 289 older adult patients with mean age of 83 years were included in the study. Serum 25(OH)D deficiency (< 50 nmol/liter) was present in 59% and hypoalbuminemia (< 3.5 g/dL) in 55% of the study patients. Low serum albumin level was associated to one, two- and five-year mortality after hospital stay (all *P* < .05). In addition, it was associated with pneumonia, dyspnea, over 13-night long stay at ward and death at ward (all *P* < .05). No associations were seen between serum 25(OH)D and LL-37 levels and disease severity, short-term clinical outcome, or long-term survival. Associations between serum 25(OH)D, albumin, and LL-37 levels and respiratory virus presence were not seen.

**Conclusions:**

Serum albumin level on admission seems to give valuable information about the patients’ general health and recovery potential in treating older adults with respiratory symptoms. Serum 25(OH)D and LL-37 had no associations with disease severity or long- and short-term prognosis among older adults hospitalized with respiratory symptoms.

**Supplementary Information:**

The online version contains supplementary material available at 10.1186/s12877-022-02836-8.

## Background

Pneumonia and other respiratory tract infections are leading causes behind infectious deaths of older adults in developed countries, and their burden of morbidity, mortality, and costs is high around the world [1–3]. All-cause mortality rate associated with respiratory viral infection increases with age and is approximately 6–7% among the over 85-year-olds [4]. Still, the clinical significance of respiratory virus infections among this group with weaning immune system remains unclear. As influenza virus tests are often the only virus test used by physicians, the role of other respiratory viruses may be underestimated in cause-of-death records [5]. At the same time, proportion of independent and vigorous older adults is increasing. By far respiratory virus diagnostics in the older adults have had low efficiency in reducing antibiotic use and the length of hospital stay [6, 7].

In addition to the important role of bone metabolism, there are vitamin D receptors in most tissues and cells in the body and multiple health benefits have been reported for vitamin D [8]. The Endocrine Society defines vitamin D deficiency as a 25-hydroxyvitamin D (25(OH)D) below 20 ng/ml (50 nmol/liter), and 25(OH)D insufficiency as a 25(OH)D of 21–29 ng/ml (52.5–72.5 nmol/liter) [8]. It is already known that serum 25(OH)D deficiency and frailty are strongly associated [9, 10]. There is some evidence that vitamin D supplement may enhance the survival of ambulatory older adults and associations have been demonstrated between serum 25(OH)D concentration and respiratory disease morbidity [11–15]. However, it is shown that serum 25(OH)D act as negative acute phase reactant and to some extent low serum values measured during acute diseases may be result of this [16].

Albumin is the most abundant plasmatic protein and its widely used as indicator of malnutrition [17]. Malnutrition again is associated with poor clinical outcome in many diseases such as cardiovascular disease [18, 19]. Hypoalbuminemia is prognostic factor for mortality among older adults [17, 20]. Low albumin levels are associated with worse recovery after acute pathologies and the inflammatory response of acute pathologies is known to decrease serum albumin level [17, 21]. Therefore, serum albumin level reflects patients inflammatory state and is associated to reduced longevity [21]. Hypoalbuminemia among adults is defined as serum albumin level below 3.5 g/dL but levels under 2.5 g/dL are often considered clinically significant [22]. Serum albumin level during hospital stay has been linked inversely to short term mortality risk [23–25]. Even more intensive treatment for community acquired pneumonia patients with low serum albumin level have been suggested [24].

LL-37 is an important human antimicrobial peptide. It is produced from precursor protein from granulocytes [26]. It acts as chemoattractant for neutrophils, monocytes, dendritic and T‐cells and is released by epithelial cell and leukocytes after infection [27]. It is expressed in epithelia of the human lung where it may have antimicrobial activity at the airway surface [28]. Serum 25(OH)D may have upregulating role of LL-37 in lung epithelium [29]. Its antimicrobial effect is suggested taking place via viral membrane damaging [30]. In vitro, LL-37 have shown antiviral activity against respiratory syncytial virus (RSV) [31]. An elevation of serum LL-37 concentration has been shown in bacterial pneumonia compared with healthy subjects [32]. There is some evidence that production of LL-37 is preserved among healthy older adults and Guo et al.have reported that LL-37 may have predictive value in 28-day survival among older adults sepsis patients [33, 34]. However, clinical studies concerning the significance of serum LL-37 levels among older adults are scarce.

As people reach higher and higher ages, the need for efficient prognostic tools and interventions against common illnesses keep growing. Among older adults, the common cold let alone pneumonia may be lethal. The aim of this study was to investigate the long-term prognostic value of serum 25(OH)D, albumin, and LL-37 levels in acute respiratory diseases among older adults. In addition, as serum 25(OH)D and albumin levels can be affected via nutrition, they may even provide interventional and preventive value in looking after our older adults. Our hypothesis was that serum 25(OH)D, albumin and LL-37 levels on admission have positive association with long-term mortality among older adults hospitalized with respiratory symptoms. We assessed one-, two- and 5-year survivals knowing that there is already some evidence of elevated 5-year mortality among patients with vitamin D deficiency [13]. We wanted to test this and see if above mentioned possibly weak and by nature fluctuating signals could be identified already with shorter one- and two- year follow-up times.

## Methods

### Study design

This prospective cohort study investigated the long-term predictive value of serum 25(OH)D, albumin, and LL-37 levels. Death was predefined as primary outcome. As secondary outcomes association between them and virus presence in nasopharynx, respiratory tract illness severity and short-term clinical outcomes in older adults hospitalized patients were studied. In addition to study samples, treatment-related laboratory diagnostics and hospital records were used for follow-up and survival analysis. STROBE criteria were respected.

### Subjects

The study was carried out in Turku City Hospital between July 2007 and April 2009 as part of a previously introduced study [35, 36]. The original study investigated the meaning of respiratory virus presence in older adults with acute respiratory disease. Consecutive, over 65 years old Turku residing patients suffering from respiratory symptoms requiring hospital admission were recruited into the original study. Patients in extremely poor condition, with severe dementia or quarantined were excluded. Information about the study was given both orally and in a written form to the patient or his/her trustee. If patient’s ability to independent decision making was deteriorated, his/her previously named trustee was approached. Serum samples were collected and used primarily for virus analysis but remaining sufficient samples were then used in this study for 25(OH)D, albumin, and LL-37 analysis. To be able to participate in the study an informed consent from the patient or his/her trustee had to be obtained. The study protocol was approved by the Ethics Committee of the Turku University Hospital, and it complies with the ethical rules for human experimentation stated in the Declaration of Helsinki [36].

### Protocol

At study entry patients or their trustees were interviewed using a standardized questionnaire, which included questions concerning the form of living before hospitalization (home, sheltered home, rest home or long-term care), the hospital unit of the patient, chronic diseases, influenza vaccination status, weight, height smoking habits and physical activity. Hospital records were reviewed for the clinical history and use of vitamin D supplements and activity of daily living (ADL) -scores. ADL-scores were defined as: 1 = bedfast, 2 = requires assistance, 3 = self-sufficient, poor motility, 4 = self-sufficient, exercises outdoors regularly. Hospital records also were used to collect dates of death during follow-up time. Study radiologist systematically analysed treatment-related chest radiographs in a blinded fashion.

### Outcomes

Death after 1, 2 and 5 years were predefined primary outcomes of this study. Secondary outcomes studied were virus presence in nasopharynx, the severity of respiratory tract disease, hospital stay length, hospital revisit and mortality during hospital stay. The severity of respiratory tract disease was estimated based on the presence of pneumonia and dyspnea and CRP and WBC levels. Cut off values 100 mg/L and 15 × 10^9^/L were used for CRP and WBC as clear and clinically usable.

### Additional definitions

Respiratory symptoms were defined as having coryza, cough, sore throat, hoarseness, or nasal stuffiness. Having one or more of the following conditions was defined as having “other diseases”: dementia, depression, diabetes, rheumatic disease or history of cancer. “Extremely poor condition” was defined as having less than a few months left to live evaluated by study physician. Interstitial infiltrate and/or lobar atelectasis in the chest radiograph was considered pneumonia after congestive heart failure as an etiology was excluded and in pneumonia episodes, the need for oxygen was considered as a sign of dyspnea. A new episode of hospital care between 2 weeks and six months from the last visit was considered a revisit. Earlier visits were considered prolonged illness and later a separate episode. The highest CRP and WBC values measured during the hospital stay were used in statistical analysis. 25(OH)D deficiency was defined as serum 25(OH)D level below 50 nmol/liter (20 ng/ml) and hypoalbuminemia as serum albumin level below 3.5 g/dL [8, 22].

### Laboratory samples

Blood samples for CRP and WBC analysis were routinely collected from all the patients as part of hospital treatment. From all patients meeting the inclusion criteria, nasopharyngeal swab samples (sterile flocked swab, 520CS01, Copan, Brescia, Italy) and serum samples were collected by study physicians within 24 h of admission. They were then stored in dry tubes in a refrigerator for a maximum of 24 h before transportation to the laboratory where they were stored at -80 °C. The swab samples were analysed at the Department of Virology, University of Turku, Turku, Finland for adenovirus, coronavirus NL63 and OC43, human bocavirus, human metapneumovirus, influenza A and B, and parainfluenza virus 1–3, RSV, rhinovirus—including rhinovirus type C—and enteroviruses (EVs). Human bocavirus infections were serologically confirmed to be acute infections at the Department of Virology, University of Helsinki, Helsinki, Finland. From these same serum samples, which also were stored at -80 °C, serum levels of 25(OH)D, albumin, and LL-37 were analysed at Massachusetts General Hospital. Serum 25(OH)D level was analyzed using immunoassay (Abbott Architect, Chicago, USA) and LL-37 was measured using ELISA (Hycult Biotech, Uden, the Netherlands), both in Massachusetts General Hospital, Boston, USA [37].

### Statistics

Patient characteristics of patients with versus without pneumonia were compared using two-sample t-test, χ2-test and Fisher exact test, as appropriate. Multivariable Cox regression analysis was used to test the associations between clinical variables and 1-, 2- and 5-year survival. Multivariable logistic regression was performed to examine the association between serum 25(OH)D, albumin, and LL-37 with clinical outcomes after adjusting for age, sex, major chronic diseases, presence of a respiratory virus and usage of vitamin D supplement. The associations of clinical variables mentioned above with serum CRP and WBC were analyzed using linear models. Natural logarithm transformed values for serum 25(OH)D, LL-37, CRP and WBC were used in the models due to positively skewed distributions. Results are expressed using adjusted odds ratios (OR), mean differences, regression coefficients and hazard ratios (HR) with their 95% confidence intervals (CI). P-values < 0.05 were considered statistically significant. Statistical analyses were performed with SAS System for Windows, version 9.4 (SAS Institute Inc., Cary, NC, USA).

## Results

### Study population

**Fig. 1:**
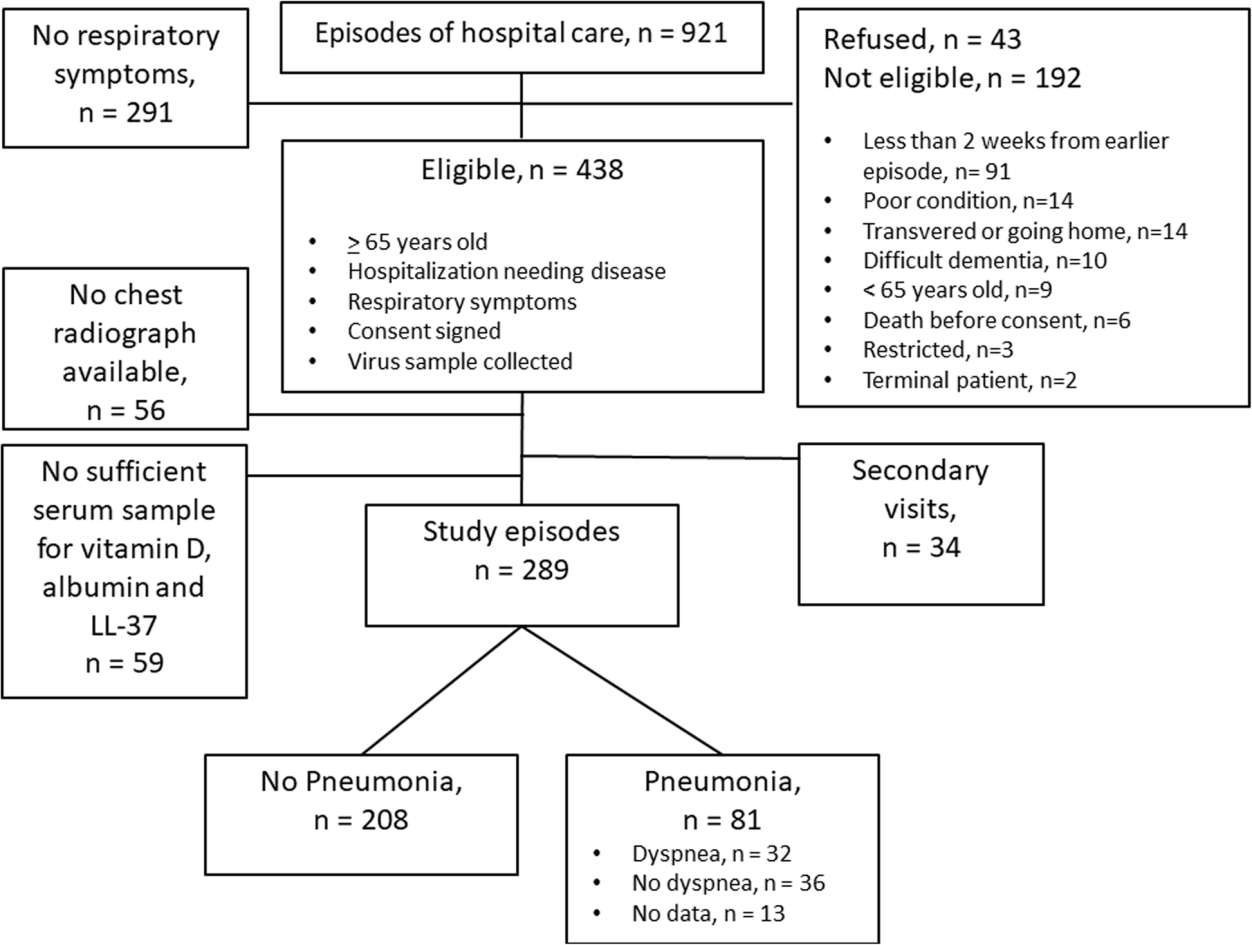
Study flow chart

As previously described, a total of 921 episodes of hospital care were screened for the study (Fig. 1) [36]. Of those 921 screened episodes of hospital care, 438 fulfilled the initial study requirements of age 65 years or over, hospitalization-needing disease, respiratory symptoms and a signed informed consent to participate in the study. Swab samples were collected from the patients of these 438 episodes. From 56 of these visits, a chest radiograph was not available and 34 episodes were secondary visits leaving 348 episodes in total. From these, a sufficient serum sample for D-vitamin, albumin and LL-37 analysis were available from 289. This study consists of these 289 episodes and patients were followed at least 5 years.

### Patient characteristics

Mean age of the patients was 83 (SD 7) years; 57% were female (Table 1). The mean age of male patients was 81 (SD 7) and female patients 84 (SD 7) (*P* = 0.001). Pneumonia was more common among male patients (54%*, P* = 0.02). Patients’ average weight was 67 kg. A diagnosis of cardiovascular disease was present in 77% and respiratory disease in 31% of the cases. Up to 71% had other disease (dementia, depression, diabetes, rheumatic disease or history of cancer). Vitamin D supplement was taken by 36% of patients. ADL scores were distributed as follows: ADL 4 with 89 (32%), ADL 3 with 107 (38%), ADL 2 with 72 (26%) and ADL 1 with 14 (5%) patients.

**Table 1:** Patient Characteristics

		**Respiratory symptoms**		
	Overall (*n* = 289)	Pneumonia (*n* = 81)	No Pneumonia (*n* = 208)	*P*
Age	83 (7)	83 (7)	83 (7)	0.82
Sex (male/female)	125/164 (43%/57%)	***44/37 (54%/46%)***	***81/127 (39%/61%)***	***0.02***
Weight	67 (17)^a^	65 (16)	68 (17)	0.16
Respiratory diseases	86/282 (31%)	27/79 (34%)	59/203 (29%)	0.40
Asthma/COPD	68/282 (24%)	21/79 (27%)	47/203 (23%)	0.55
Other lung disease	27/282 (10%)	7/79 (9%)	20/203 (10%)	0.80
Cardiovascular diseases	216/282 (77%)	60/79 (76%)	156/203 (77%)	0.87
Other diseases	201/282 (71%)	54/79 (68%)	147/203 (72%)	0.50
Dementia	60/282 (21%)	17/79 (22%)	43/203 (21%)	0.95
Depression	37/289 (13%)	11/81 (14%)	26/208 (13%)	0.81
Rheumatic disease	81/282 (29%)	20/79 (25%)	61/203 (29%)	0.43
Diabetes mellitus				0.65
Type 1	2/282 (1%)	0/79 (0%)	2/203 (1.0%)	
Type 2	62/282 (22%)	15/79 (17%)	47/203 (23%)	
Cancer	66/282 (23%)	15/79 (19%)	51/203 (25%)	0.67
Smoking	37/282 (17%)	15/64 (23%)	22/155 (14%)	0.09
Vitamin D supplement	103/289 (36%)	29/81 (36%)	74/208 (36%)	0.97
ADL-score 4	89 (32%)^b^	27 (34%)	62 (31%)	
ADL-score 3	107 (38%)	30 (38%)	77 (38%)	
ADL-score 2	72 (26%)	19 (24%)	53 (26%)	
ADL-score 1	14 (5%)	3 (5%)	11 (5%)	
Vaccination	112/282 (40%)	34/79 (43%)	78/203 (38%)	0.48

Abbreviations: COPD, chronic obstructive pulmonary disease, ADL, activity of daily living

Data expressed as n (%) or mean (SD)

Two-sample *t*-test, χ^2^-test and Fisher exact test were used

Significant values are shown bold and italic

*COPD* chronic obstructive pulmonary disease

^a^Weight known from 258 patients

^b^ADL score known from 282 patients

Statistically significant values bolded and in italic

### Association between serum 25(OH)D level and clinical outcomes

Serum 25(OH)D level nor the use of vitamin D supplement was associated with one-, two- or five-year survival in multivariable Cox regression survival analysis (Table 2). Mean serum 25(OH)D level among study patients was 47 nmol/l (SD 22 nmol/l). A majority, 59%, of the study patients had 25(OH)D level below 50 nmol/l. In logistic regression analysis, serum 25(OH)D level was not associated with clinical outcomes studied (Table 3). In linear models, association between serum maximum CRP and WBC values with serum 25(OH)D level could not be shown either (Table 4). Difference could not be shown in numbers of serum 25(OH)D insufficiency between patients with (63%) and without (58%) pneumonia *(P* = 0.46).

**Table 2:** Association between clinical variables and survival (*n* = 282)

	1-year survival		2-year survival		5-year survival	
	Adjusted HR (95% CI)	*P*	Adjusted HR (95% CI)	*P*	Adjusted HR (95% CI)	*P*
**Ln 25(OH)D**	1.06 (0.64, 1.75)	0.83	1.19 (0.79, 1.78)	0.40	1.09 (0.79, 1.51)	0.61
**Albumin**	***0.19 (0.11, 0.32)***	***<0.0001***	***0.34 (0.22, 0.53)***	***<.0001***	***0.54 (0.38, 0.77)***	***0.0005***
**Ln LL-37**	0.92 (0.49, 1.72)	0.80	1.10 (0.70, 1.75)	0.67	1.19 (0.83, 1.72)	0.35
**Age**	***1.06 (1.03, 1.10)***	***0.0007***	***1.05 (1.02, 1.08)***	***0.0007***	***1.05 (1.03, 1.07)***	***<.0001***
**Female sex**	0.97 (0.61, 1.57)	0.91	0.70 (0.49, 1.02)	0.06	***0.56 (0.42, 0.76)***	***0.0002***
**Respiratory disease**	1.54 (0.94, 2.51)	0.09	1.37 (0.93, 2.01)	0.11	1.27 (0.93, 1.73)	0.14
**Cardiovascular disease**	1.04 (0.59, 1.85)	0.88	1.08 (0.69, 1.69)	0.73	1.11 (0.78, 1.57)	0.57
**Other disease**	1.79 (0.99, 3.24)	0.06	***1.99 (1.26, 3.15)***	***0.003***	***1.92 (1.37, 2.70)***	***0.0002***
**Vitamin D supplement**	0.95 (0.56, 1.61)	0.85	1.11 (0.74, 1.68)	0.61	1.13 (0.81, 1.57)	0.48
**Respiratory virus**	0.93 (0.58, 1.50)	0.77	0.92 (0.64, 1.33)	0.65	***0.68 (0.50, 0.92)***	***0.01***

Multivariable Cox regression survival analysis was used

*HR* Hazard Ratio

*CI* Confidence interval

*Ln 25(OH)D* natural logarithm of serum 25(OH)D concentration

*Ln LL-37* natural logarithm of serum LL-37 concentration

*DvitSupp* Usage of 25(OH)D supplement

Statistically significant values bolded and in italic

**Table 3:** Association between serum 25(OH)D, LL-37 and albumin with clinical outcomes

**Outcome**		**Ln 25(OH)D** **Adjusted**		**Ln LL-37** **Adjusted**		**Albumin** **Adjusted**	
	**n**	**OR (95% CI)**	***P***	**OR (95% CI)**	***P***	**OR (95% CI)**	***P***
**Pneumonia**	282	1.10 (0.59, 2.04)	0.76	1.15 (0.57, 2.31)	0.69	***0.43 (0.22, 0.82)***	***0.01***
**Dyspnea**	244	0.72 (0.39, 1.33)	0.29	1.30 (0.60, 2.78)	0.51	***0.47 (0.24, 0.90)***	***0.02***
**Death at ward**	282	0.86 (0.27, 2.69)	0.79	0.91 (0.22, 3.77)	0.90	***0.06 (0.02, 0.24)***	** < *.0001***
**Over 13 nights at ward**	282	1.11 (0.61, 2.03)	0.73	1.39 (0.70, 2.76)	0.35	***0.41 (0.22, 0.77)***	***0.006***
**Revisit**	282	1.43 (0.80, 2.58)	0.23	0.82 (0.43, 1.57)	0.54	1.02 (0.56, 1.88)	0.95
**CRP over 100 mg/L**	282	0.73 (0.40, 1.34)	0.31	***3.89 (1.79, 8.45)***	***0.0006***	***0.22 (0.11, 0.42)***	** < *.0001***
**WBC over 15 E9/L**	279	0.78 (0.38, 1.63)	0.51	1.84 (0.79, 4.32)	0.16	0.52 (0.24 1.12)	0.09

Multivariable logistic regression analysis adjusted with age, sex, existing of respiratory, cardiovascular, or other disease, presence of a respiratory virus and usage of vitamin D supplement was used

*OR* Odds Ratio

*CI* Confidence interval

*Ln 25(OH)D* natural logarithm of serum 25(OH)D concentration

*Ln LL-37* natural logarithm of serum LL-37 concentration

Statistically significant values bolded and in italic

**Table 4:** Association of clinical variables with serum CRP and WBC values

	**Ln CRP (*n* = 282)**		**Ln WBC (*n* = 279)**	
	Adjusted estimate (95% CI)	*P*	Adjusted estimate (95% CI)	*P*
**Ln 25(OH)D**	-0.16 (-0.41, 0.10)	0.24	-0.052 (-0.195, 0.093)	0.48
**Albumin**	***-0.75 (-1.03, -0.48)***	** < *0.0001***	-0.13 (-0.28, 0.02)	0.10
**Ln LL-37**	***0.38 (0.09, 0.68)***	***0.01***	***0.24 (0.08, 0.40)***	***0.004***
**Age**	-0.0090 (-0.0259, 0.0080)	0.30	-0.0044 (-0.0138, 0.0051)	0.36
**Female sex**	***-0.30 (-0.55, -0.06)***	***0.01***	0.0034 (-0.1310, 0.1378)	0.96
**Respiratory disease**	0.052 (-0.199, 0.303)	0.69	0.056 (-0.084, 0.196)	0.43
**Cardiovascular disease**	0.050 (-0.222, 0.322)	0.72	-0.018 (-0.168, 0.133)	0.82
**Other disease**	0.034 (-0.222, 0.290)	0.79	0.059 (-0.084, 0.201)	0.42
**Vitamin D supplement**	0.077 (-0.193, 0.348)	0.57	0.11 (-0.04, 0.26)	0.14
**Respiratory virus**	-0.0020 (-0.2352, 0.2311)	0.99	-0.12 (-0.25, 0.01)	0.06

Linear model was used

Estimates are expressed as regression coefficient (95% confidence interval) for continuous variables (Ln 25(OH)D, Albumin, Ln LL-37 and Age) and as mean difference (95% confidence interval) for categorical variables

*Abbreviations:*

*Ln CRP* natural logarithm of the maximum value of serum C-reactive protein

*Ln 25(OH)D* natural logarithm of serum 25(OH)D concentration

*Ln LL-37* natural logarithm of serum LL-37 concentration

*Ln WBC* natural logarithm of serum maximum white blood cell count

Statistically significant values bolded and in italic

### Association between LL-37 and clinical outcomes

Mean serum LL-37 level among study patients was 38 ng/ml (SD 32 ng/ml). There was no association between serum LL-37 level and survival (Table 2). In logistic regression analysis, serum LL-37 concentration showed association with serum CRP values over 100 mg/L *(P* = 0.0006, Table 3). An association was also seen in linear models between serum LL-37 and CRP and WBC levels (respectively, *P* = 0.01 and *P* = 0.004, Table 4). Association to other clinical outcomes was not seen.

### Association between albumin and clinical outcomes

Mean serum albumin level among study patients was 3.4 g/dl (SD 0.4 g/dl). In total, 159 (55%) of the study patients had hypoalbuminemia. Low serum albumin level and high age were associated with higher one-year mortality in multivariable Cox regression analysis (respectively, *P* < 0.0001 and *P* = 0.0007, Table 2). In addition of serum albumin level and age (*P* < 0.0001 and *P* = 0.0007), the presence of other disease (*P* = 0.003) was associated with higher two-year mortality in the multivariable model. Respectively, the variables mentioned above, sex and presence of a respiratory virus were associated with 5-year mortality (respectively, *P* = 0.0002 and *P* = 0.01). Female sex and virus presence were associated with better survival.

Hypoalbuminemia was more common among patients with pneumonia (72%) than among patients without pneumonia (49%) *(P* = 0.0004). Moreover, low serum albumin level was associated with pneumonia, dyspnea, over 13-night long stay at ward, death at ward and serum CRP value over 100 mg/L in multivariable logistic regression analysis *(P* = 0.01, *P* = 0.02, *P* = 0.006, *P* < 0.0001 and *P* < 0.0001, respectively, Table 3). However, albumin was not associated with white blood cell counts over 15 and not with hospital revisit. In predicting pneumonia, in addition to serum albumin level, also sex was statistically significant in the model (*P* = 0.049, Table 5). Similarly, in addition to serum albumin level, presence of respiratory disease was statistically significant in the model predicting dyspnea (OR = 2.79, 95% CI 1.54–5.08, *P* = 0.0008). Age was statistically significant variable in the model predicting death at ward together with albumin (OR = 1.09, 95 CI 1.00–1.17, *P* = 0.04).

**Table 5:** Association of clinical variables with pneumonia and virus presence (*n* = 282)

	**Pneumonia**		**Virus presence**			
	**Adjusted OR (95% CI)**	***P***	**Adjusted OR (95% CI)**	***P***		
**Ln 25(OH)D**	1.06 (0.57, 2.0)	0.86	1.4 (0.82, 2.5)	0.21		
**Albumin**	***0.41 (0.22, 0.79)***	***0.007***	1.4 (0.80, 2.6)	0.22		
**Ln LL-37**	1.2 (0.59, 2.4)	0.62	0.72 (0.38, 1.4)	0.31		
**Vitamin D supplement**	0.97 (0.51, 1.8)	0.92	0.88 (0.49, 1.6)	0.68		
**Age**	1.0 (0.97, 1.1)	0.55	0.99 (0.95, 1.0)	0.57		
**Female sex**	***0.57 (0.32, 1.0)***	***0.049***	0.88 (0.52, 1.5)	0.62		
**Respiratory disease**	1.2 (0.66, 2.1)	0.58	0.73 (0.43, 1.3)	0.27		
**Cardiovascular disease**	0.94 (0.49, 1.8)	0.84	1.0 (0.56, 1.8)	0.98		
**Other disease**	0.83 (0.45, 1.5)	0.54	1.5 (0.87, 2.7)	0.14		
	**Influenza**		**Rhinovirus**		**RSV**	
	**Adjusted OR (95% CI)**	***P***	**Adjusted OR (95% CI)**	***P***	**Adjusted OR (95% CI)**	***P***
**Ln 25(OH)D**	0.98 (0.37, 2.6)	0.97	1.4 (0.57, 3.5)	0.46	2.1(0.68, 6.7)	0.20
**Albumin**	2.6 (0.92, 7.4)	0.07	0.74 (0.30, 1.9)	0.52	1.3 (0.41, 4.2)	0.65
**Ln LL-37**	0.48 (0.17, 1.4)	0.18	0.98 (0.37, 2.6)	0.97	1.1 (0.32, 3.7)	0.89
**Vitamin D supplement**	0.50 (0.16, 1.6)	0.23	1.4 (0.56, 3.4)	0.48	0.42 (0.13, 1.4)	0.15
**Age**	1.0 (0.95, 1.1)	0.82	0.99 (0.93, 1.1)	0.74	1.0 (0.95, 1.1)	0.65
**Female sex**	0.55 (0.23, 1.3)	0.18	0.97 (0.43, 2.2)	0.94	2.0 (0.68, 5.7)	0.21
**Respiratory disease**	1.2 (0.51, 3.0)	0.64	1.0 (0.43, 2.4)	0.97	0.89 (0.30, 2.7)	0.83
**Cardiovascular disease**	1.5 (0.53, 4.5)	0.42	0.66 (0.27, 1.6)	0.35	0.76 (0.25, 2.3)	0.62
**Other disease**	1.1 (0.44, 2.8)	0.83	1.9 (0.67, 5.3)	0.23	0.67 (0.25, 1.9)	0.45

Multivariable logistic regression was used

*OR* Odds Ratio

*CI* Confidence interval

*Ln 25(OH)D* natural logarithm of serum 25(OH)D concentration

*Ln LL-37* natural logarithm of serum LL-37 concentration

*DvitSupp* Usage of Vitamin D supplement

Statistically significant values bolded and in italic

## Discussion

This cohort study of 289 older adult patients (mean age 83 years) has four main findings. First, hypoalbuminemia was associated up to five-year mortality after hospital stay. Second, 25(OH)D deficiency and hypoalbuminemia were relatively common; they were present in 59% and 55% of the study patients, respectively. Third, hypoalbuminemia was associated with many adverse outcomes including pneumonia, dyspnea, over 13-night long stay at ward and death at ward. Fourth, against our expectations, no associations were seen between serum 25(OH)D and LL-37 levels and long- and short-term clinical outcomes or disease severity. Moreover, associations between serum 25(OH)D, albumin or LL-37 levels and respiratory virus presences were not seen.

More than a half of the study patients suffered from 25(OH)D deficiency in our study. In a Norwegian study with 241 patient cohort of community acquired-pneumonia (CAP) survivors, there was a high prevalence of 25(OH)D deficiency and inadequacy among hospitalized adults with CAP [13]. We did not see difference between serum 25(OH)D levels among patients with and without pneumonia. The Norwegian study also suggested that 25(OH)D deficiency could be associated with an increased risk of long-term mortality. In our study, serum 25(OH)D level or vitamin D supplement usage was not associated with one-, two- or five-year survival in contrast to finding in 1834 patient study performed in China [38]. In a South Korean study, serum 25(OH)D concentration and 28-day all-cause mortality were linked together among 797 pneumonia patients with mean age 68.1 years [12]. In our study short-term outcomes; death at ward, revisit or over 13 nights hospital stay did not associate to serum 25(OH)D level.

As hypoalbuminemia among adults is defined as serum albumin level below 3.5 g/dL, the majority of our study patients suffered from hypoalbuminemia. Hypoalbuminemia is known to be a prognostic factor for mortality among older adults and it is associated to worse recovery after acute pathologies [17, 20, 25]. It is also known that serum albumin level decreases in infections [21]. Our study strengthens both these ideas. Hedlund states in his study that a low serum albumin concentration was the most important factor independently associated with fatal disease in community-acquired pneumonia hospitalizations. He underlines that CAP patients with low serum albumin level should therefore be observed and treated more intensively [24]. Our study confirms this idea. We found that low serum albumin level on hospital admission was associated to pneumonia, disease severity based on elevation of CRP level and was in fact a clear prognostic factor during one-, two- and five-year surveillance time. Age together with serum albumin level on admission were two independent factors that were prognostic regarding mortality from one to five years. Two-year mortality was associated in addition to the presence of other disease. Five-year mortality was already associated with many factors; serum albumin, age, sex, other disease and the presence of respiratory virus. Interestingly the presence of a respiratory virus on hospital admission was associated to better 5-year survival. This could be explained by the fact that respiratory viruses typically cause self-limiting illnesses and being present on admission, more serious reasons for hospitalization may have been more likely absent.

We saw no association between LL-37 and long- or short-term clinical outcomes other that disease severity describing CRP and WBC. Guo et al. reported that among older adults sepsis patients LL-37 may have predictive value in 28-day survival [34]. We didn’t see this kind of association. Previously, antiviral activity of LL-37 against RSV have been shown in vitro [31]. In our study, serum LL-37 level was not associated with virus presence overall or with most prevalent single viruses, influenza virus, rhinovirus, or RSV. Majewski et al. reported elevation of serum LL-37 concentration in bacterial pneumonia compared with healthy subjects [32]. Association between serum LL-37 level and pneumonia was not seen in our study. However, serum LL-37 level was associated to serum CRP level and WBC. Castañeda-Delgado et al. have suggested in their study that production of LL-37 is preserved among healthy older adults [33]. We confirm this with our study patients, older adult patients with acute respiratory disease. There was no association between LL-37 level and age in our study and therefore the production could be considered preserved with increasing age.

The strengths of our study include prospective design, relatively large sample size and long follow-up time. Also, pneumonia was radiologically confirmed. The study focused on serum 25(OH)D, albumin, and LL-37 levels in relations to each other and other common clinical factors, which bring the results closer to the everyday practical work. There are some limitations as well. One important limitation of the study is that it does not take into account the seasonal circadian variation of vitamin D and more detailed nutritional status was not measured. Patients’ height was managed to collect from only a few patients and for this reason it had to be left out of the study. In addition, acute illness effect on serum 25(OH)D and albumin levels as acute phase reactants and therefore the results from this study may not be directly usable in treating patients without acute illness. As the study observed hospitalization-requiring episodes among geriatric patients with multimorbidity, the results cannot be generalized as such to treating outpatients.

## Conclusions

Serum albumin level on admission seem to give valuable information about the patients’ long-term prognosis, general health and recovery potential in treating older adults with respiratory symptoms. At the same time serum 25(OH)D and LL-37 levels don’t seem to have long- or short-term prognostic value at mean level of 47 nmol/l and 38 ng/ml respectively. Our finding underlines the importance of good nutritional status of the older adults.

## Supplementary Information

**Additional file 1. **Standard clinical questionnaire.

## Data Availability

The datasets used and/or analysed during the current study are available from the corresponding author on reasonable request.

## Abbreviations

CI: Confidence interval
COPD: Chronic obstructive pulmonary disease
CRP: C-reactive protein
OR: Odds ratio
RSV: Respiratory syncytial virus
SD: Standard deviation
WBC: White blood cell count
25(OH)D: 25-Hydroxy25(OH)D
