# Supplementary material for: The long-term prognostic value of serum 25(OH)D, albumin, and LL-37 levels in acute respiratory diseases among older adults
Source: BMC Geriatr. 2022 Feb 21;22:146. doi: 10.1186/s12877-022-02836-8 (PMC8860370; doi:10.1186/s12877-022-02836-8)
Supplement: Supplementary file 1 — Additional file 1. Standard clinical questionnaire. [file 12877_2022_2836_MOESM1_ESM.docx]

**Potilaan nimi __________________________________________________________ Henkilötunnus ___________________________**

**Tuloarvio:**

Asumismuoto ennen sairaalaan tuloa: koti __, palveluasunto __, vanhainkoti _, pitkäaikaissairaanhoito __

Mistä tulee: tk päiv__, ksh__, TYKS ea __, sairaalasiirto __

Paino _______ kg, pituus ____ cm

Pitkäaikaissairaudet:  keuhkosairaus, mikä/mitkä ______________________________________________________________________

 sydän- ja verenkiertoelimistön sairaus, mikä/mitkä ___________________________________________________

 diabetes, mikä tyyppi ja kuinka kauan _____________________________________________________________

 sidekudostauti/reuma, mikä/mitkä ________________________________________________________________

 syöpä, mikä/mitkä _____________________________________________________________________________

 immuunipuutos, syy/mikä/mitkä _________________________________________________________________

 muu sairaus, mikä/mitkä ________________________________________________________________________

Lääkitys:________________________________________________________________________________________________________

_______________________________________________________________________________________________________________

_______________________________________________________________________________________________________________

Influenssa ja muut rokotukset:_______________________________________________________________________________________

Tupakointi:  kyllä,  ei koskaan,  lopettanut, kuinka monta vuotta sitten: ___________________________________________________

Fyysinen aktiivisuus:  vuodepot.,  avustettava,  omatoiminen, liikkuminen vähäistä,  omatoiminen, ulkoilee säännöllisesti

Ravitsemustila/nestetasapaino: MNA (oma kaavake)

Dementiaseula: MMSE (oma kaavake)

Kontakti flunssaisiin ihmisiin 2 vk ajan ennen sairaalaan tuloa:  kyllä,  ei,  ei tiedossa

Kontakti lapsiin 2 vk ajan ennen sairaalaan tuloa:  kyllä,  ei,  ei tiedossa

Syy sairaalahoitoon? ______________________________________________________________________________________________

**Patients name ________________________________________________________Social security number ___________________________**

**Entry evaluation:**

Form of living before hospitalization: home __, service home __, rest home _, institution for chronically ill patients __

From where arriving: health care centers emergency room__, home nursing__, Turku university hospital emergency room __, transfer between hospitals __

Weight_______ kg, Height ____ cm

Chronic illnesses:  pulmonary disease, what ______________________________________________________________________

 cardiovascular disease, what ____________________________________________________________________

 Diabetes, type and since when ___________________________________________________________________

 connective tissue / rheumatic disease, what _________________________________________________________

 cancer, type _____________________________________________________________________________

 immunodeficiency, reason/what type _________________________________________________________________

 other illness, what ________________________________________________________________________

Medication: ________________________________________________________________________________________________________

_______________________________________________________________________________________________________________

_______________________________________________________________________________________________________________

Influenza and other vaccinations: _______________________________________________________________________________________

Smoking:  yes,  never,  quitted, how many years ago: _________________________________________________________________

Physical activity:  bedridden.,  assistance needing,  self-sufficient, low physical activity,  self-sufficient, daily outdoor life

Nutrition/fluid balance: MNA (separate form)

Dementia screenin: MMSE (separat form)

Interaction with individuals with flu 2 weeks pior hospitalization:  yes,  no,  not known

Interaction with children 2 weeks prior hospitalization:  yes,  no,  not known

Reason to hospitalization? ______________________________________________________________________________________________
